# COVID 19 - Clinical Picture in the Elderly Population: A Qualitative Systematic Review

**DOI:** 10.14336/AD.2020.0620

**Published:** 2020-07-23

**Authors:** Agnieszka Neumann-Podczaska, Salwan R Al-Saad, Lukasz M Karbowski, Michal Chojnicki, Slawomir Tobis, Katarzyna Wieczorowska-Tobis

**Affiliations:** ^1^Geriatrics Unit, Department of Palliative Medicine, Poznan University of Medical Sciences, Poznan, Poland.; ^2^Department of Biology and Environmental Protection, Poznan University of Medical Sciences, Poznan, Poland.; ^3^Department of Infectious Diseases, Jozef Strus Hospital, Poznan, Poland; ^4^Occupational Therapy Unit, Geriatrics and Gerontology, Poznan University of Medical Sciences, Poznan, Poland; ^5^Geriatric Outpatient Clinic, University Hospital of Lord’s Transfiguration, Poznan, Poland.

**Keywords:** COVID-19, SARS-CoV-2, elderly, older individuals, clinical features

## Abstract

The SARS-CoV-2 tendency to affect the older individuals more severely, raises the need for a concise summary isolating this age population. Analysis of clinical features in light of most recently published data allows for improved understanding, and better clinical judgement. A thorough search was performed to collect all articles published from 1st of January to 1st of June 2020, using the keywords *COVID-19* and *SARS-CoV-2* followed by the generic terms *elderly*, *older adults* or *older individuals*. The quality assessment of studies and findings was performed by an adaptation of the STROBE statement and CERQual approach. Excluding duplicates, a total of 1598 articles were screened, of which 20 studies were included in the final analysis, pertaining to 4965 older COVID-19 patients (≥60 years old). Variety in symptoms was observed, with fever, cough, dyspnea, fatigue, or sputum production being the most common. Prominent changes in laboratory findings consistently indicated lymphopenia and inflammation and in some cases organ damage. Radiological examination reveals ground glass opacities with occasional consolidations, bilaterally, with a possible peripheral tendency. An evident fraction of the elderly population (25.7%) developed renal injury or impairment as a complication. Roughly 71.4% of the older adults require supplementary oxygen, while invasive mechanical ventilation was required in almost a third of the reported hospitalized older individuals. In this review, death occurred in 20.0% of total patients with a recorded outcome (907/4531). Variability in confidence of findings is documented. Variety in symptom presentation is to be expected, and abnormalities in laboratory findings are present. Risk for mortality is evident, and attention to the need for supplementary oxygen and possible mechanical ventilation is advised. Further data is required isolating this age population. Presented literature may allow for the construction of better predictive models of COVID-19 in older populations.

In December 2019, the Chinese health officials informed the World Health Organization (WHO) of the emergence of an unknown pneumonia-like illness, originating from the Hubei province, Wuhan. Following the appearance of numerous cases worldwide, the WHO, in March 2020, announced that the coronavirus disease (COVID-19), caused by the Severe Acute Respiratory Syndrome Coronavirus-2 (SARS CoV-2), is a global pandemic of imminent danger.

The elderly and geriatric population are amongst the highest risk patients for severe complications as a result of COVID-19 [1,2,3,4,5]. Early data has shown that individuals > 59 years of age are estimated to be 5 times more likely to die following the onset of COVID-19 symptoms as compared to those between the ages of 30-59 [6]. Early recognition of at-risk older patients and awareness of potential atypical clinical presentation in the older population becomes vital to circumvent lethal complications.

To date, the collection of clinical parameters and comprehension of the COVID-19 infection and how it may present in older individuals is limited. Such understanding becomes key in guiding clinical judgement and may allow for better preparation of health care professionals yet to face the disease. In the following study, we constructed a systematic review to concisely summarize the clinical features, comorbidities, radiological/laboratory findings, and outcomes in the older adults. Data for the current therapeutic approaches currently being used was also collected.

## METHODS

### Search Strategy and Criteria

Systematic search was performed for articles published between January 1, 2020, and June 1, 2020, using the primary databases PubMed and ScienceDirect. To ensure greater coverage of literature, complementary databases such as Wiley Online Library and Google Scholar were also used, with no restriction to language. The keywords *COVID-19* OR *SARS CoV 2* were followed by each of *elderly* OR *older adults* OR *older individuals* independently during the search of literature. Studies were also collected from brief screening of reference lists of high relevance articles. Due to large search results and overlap of studies, the complementary databases were screened with more strict search settings (such as the necessity of “covid 19” or “sars cov 2” presence in the title and presence of at least one of “elderly” “older adult” “older individuals” in the article text or abstract). Such limits were not implemented in the search of primary databases (PubMed and ScienceDirect).

### Selection and Eligibility

Following removal of duplicates and to ensure quality of standard selection, the two researchers who completed the literature search performed an initial screening of the collected articles independently [7]. Papers such as guidelines, public health advice, psychological studies, surveys, genetic and viral studies (oriented to pathogenesis and mechanisms or other serotypes) were all excluded based on brief view of the paper, abstract, and title. Once relevant articles were isolated, the two reviewers further independently assessed full text eligibility based on few major, yet strict, criteria.

Only older individuals (≥60 years old) with confirmed SARS-CoV-2 infection were of interest in this review, hence any study that did not clearly separate the data according to this age bracket was excluded. Calculations were made to ensure that the interquartile range (IQR), standard deviations (SD), and ranges mentioned in any study indeed fit the age criteria. Study populations with IQR, SD, or min/max range that include patients of <60 years of age were excluded, unless the paper explicitly stated that the participants were ≥60 years old. Retrospective studies (descriptive, case reports, case series, case-control, cross sectional studies) and cohort studies were included, except in cases where unique conditions were considered to possibly influence the disease presentation and data. This includes patients with extremely unique underlying diseases, following extensive medical treatment for certain comorbidities, or clinical trials of drugs. Any studies with only few reported data were considered on an individual basis and excluded or included accordingly. Considering the descriptive nature of this review, studies with larger sample sizes were deemed more valuable, therefore studies with only ≤ 2 older patients were excluded. Review articles and other systematic reviews were assessed for reference list relevance, however the review articles themselves were excluded. Results that may disproportionately sway the incidence rates due to sums of patients irreflective of the general population were also isolated. Any disparities concerning the studies were settled by means of discussion and eventual consensus between all the reviewers.

### Retrieval Strategy and Data Extraction

Initial search of databases revolved around identifying and isolating the number of search findings, followed by the exclusion of duplicates. Once this was established, two independent reviewers screened the studies for general relevance to the review topic. Studies with unrelated subject matter or studies with indirect relevance (incorrect study population) were then excluded. Next, the remaining studies were assessed more thoroughly for eligibility. At this stage, closer assessment for inclusion/exclusion criteria was performed (criteria mentioned in “Selection and Eligibility”). Discussion and eventual consensus were reached between reviewers regarding the final included studies.

Once the relevant studies were isolated, the two reviewers extracted the data independently into a standardized form with the following subheadings: paper information (such as publication date, number of patients, country, and gender), symptoms, comorbidities, laboratory findings, radiographic findings, complications, treatment, and outcome. To better reflect current literature, all the data and all the variables accessible from the studies concerning COVID-19 patients ≥60 years old was extracted into the forms. If a study had relevant data merged with age groups <60 years old, that data was not included. Cross checking and discussion was then performed concerning the 2 forms, with 3rd reviewer involvement in occasional differences. Consensus was reached regarding variables to include within subheadings.

All the data is relative to date of publication, no follow up on cases was performed. Concerning data synthesis in tables, all the subheadings, besides laboratory findings, included incidence data in the form of a percentage (%), with or without the number of patients. The use of incidence percentage to describe the data facilitated easier visualization of patterns within subheadings. Percentage of involved patients was calculated based on total patients ≥60 in the respective study. When finding a combined percentage for multiple studies, incidence was added and divided by the total study populations (≥60 years old) included. As for case series, where results of patients were presented individually, median and interquartile range (IQR) was calculated for the pertinent subpopulation.

### Quality Assessment

The quality assessment of the individual studies was performed using an adaptation of the STROBE (Strengthening the Reporting of Observational Studies in Epidemiology) statement [8]. The elements scrutinized the most included study population demographic and characteristics, eligibility, methodology and methods of attaining data, duration of follow up, possible source of bias (particularly selection bias), and incomplete or missing data.

Implementing the CERQual approach [9], the review findings were then given a transparent confidence score based on combined qualitative assessment of contributing studies, taking into consideration any relevant limitations. This allows for better judgement of findings based on allocated confidence. Components analyzed include methodological limitations, coherence of results, adequacy and sufficiency of data, as well as aligned relevance to review topic. Four levels of confidence in findings were utilized: High (*highly likely* that review findings is a reasonable representation of phenomena), Moderate (*likely* that review findings is a reasonable representation of phenomena), Low (*it is possible* that review findings is a reasonable representation of phenomena), and Very Low (*it is not clear* whether the review finding is a reasonable representation of phenomena).

## RESULTS

**Figure 1.**
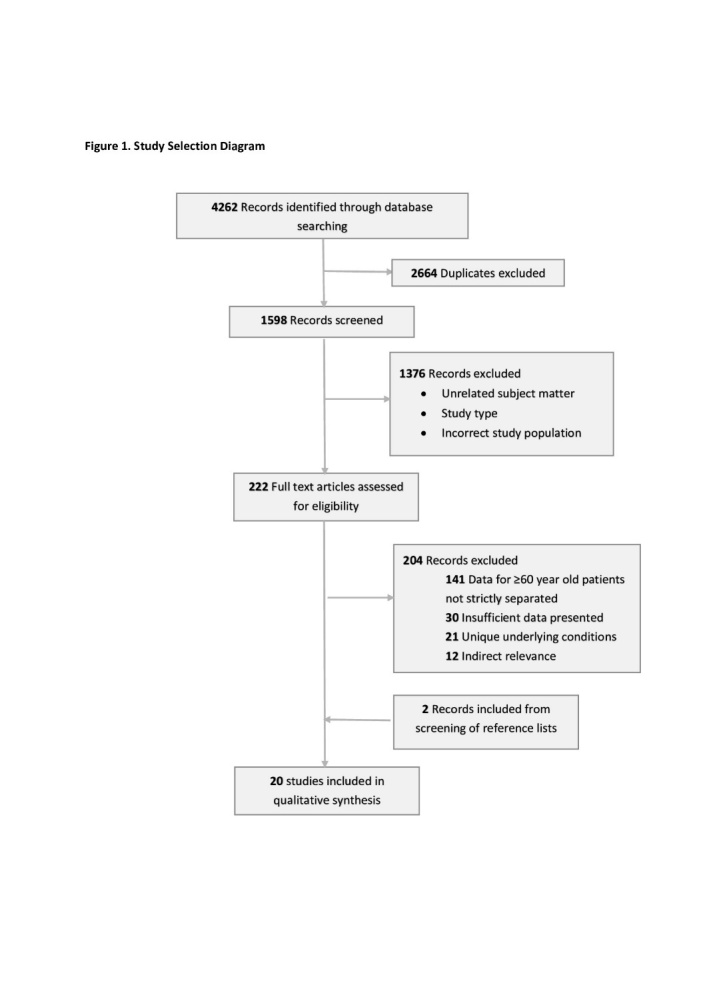
Study Selection Diagram.

Initial search of databases showed 4262 findings, which yielded 1598 articles when excluding the duplicates. Of those articles, 222 were considered relevant based on screening of title, abstract and brief view of content. 1376 studies were excluded due to unrelated subject matter (psychological studies, surveys, virological/gene-related studies, mechanisms and pathogenesis), study type (guidelines, reviews), or incorrect study population (children, adolescence, adults <60 years old). Following individual assessment of the 222 studies, twenty met the inclusion criteria, in which 2 were retrieved from screening of reference lists. All relevant studies were retrospective in nature. Most studies were excluded due to improper separation of COVID-19 patients ≥60 years old or insufficient data presentation (Fig. 1).

**Table 1:** Symptoms, Comorbidities, Radiological Findings, and Outcome.

	Wang L et al. [10]	Zhu X et al. [11]	Lian J et al. [12]	^*^Cheng B et al. [13]	^*^Cheng B et al. [13]	Chen T et al. [14]	Liu K et al. [15]	Godaert L et al. [16]	Liu Y et al. [17]	Mi B et al. [18]
**Publication Month - 2020**	Mar	Mar	Mar	Apr	Apr	Apr	Mar	Apr	Mar	Apr
**Country**	China	China	China	China	China	China	China	France	China	China
**No. Patients ≥60 Years Old**	339	114	136	67	51	55	18	17	7	6
**Age**	>60	>70	≥60	≥60	≥60	≥65	≥60	>67	>61	>75
**Male No.**	166	67	58	22	31	34	12	8	4	2
**Symptoms^+^ (%)**										
	(92) Fev	(82) Fev	(85) Fev	(74) Cgh	(83) Fev	(95) Fev	(78) Fev	(77) Fev	(100) Fev	(84) Fev
	(53) Cgh	(60) Cgh	(63) Cgh	(60) Fev	(64) Cgh	(70) Cgh	(33) Cgh	(70) Cgh	(100) Cgh	(84) Cgh
	(41) Dysp	(46) Dysp	(36) SP	(57) CT	(64) CT	(64) CT	(33) SP	(65) Dysp	(58) Chi	(67) Dysp
	(40) Ft	(45) Ft	(18) Ft	(48) Ft/Mya	(56) Ft/Mya	(60) Dysp	(17) N/V	(59) Ft/Mya	(43) Ft/Mya	(67) Ft/Mya
	(28) Anx	(33) SP	(15) Mya	(36) SP	(48) N/V	(20) Mya	(11) CT	(53) Del	(14) Dh	(50) ST
	(28) SP	(9) Dh	(13) ST	(21) N/V	(26) SP	(10) Ft	(11) Dysp	(36) Dh	(14) N/V	(33) Dizz
**Others**	(26) CT (13) Dh	-	(13) Dysp (8) Dh (6) Hdc	(14) Hdc (14) Diz	(12) Hdc (12) Diz (6) Dh (10) Con (6) AG	(10) Anx (6) Hdc (6) Dh (6) AP	(11) Ft/Mya (6) NC	(18) SP	-	(17) Hdc (17) N/V (17) NC (17) CP (17) AP
**≤5 %**	Mya, Hdc, N/V, Diz, Pha	Mya, N, V, Pha	NC, Hp	Dh, AG	-	N/V, CP	-	-	-	-
**Comorbidities^++^ (%)**										
	(41) HTN	(54) CVD	(39) HTN	(59) HTN	(50) HTN	(39) HTN	(46) Smo	-	(58) CVD	(50) OP
	(16) DM	(22) CLD	(18) DM	(17) DM	(32) DM	(22) DM	(28) HTN	-	(43) HTN	(33) HTN
	(16) CVD	(22) PS	(6) Smo	(17) CVD	(24) CVD	(20) CVD	(17) DM	-	(29) CRD	(17) DM
	(7) CLD	(20) CBD	-	(10) CLD	(12) CLD	(15) CBD	(17) CVD	-	(14) DM	(17) CLD
	(7) CBD	(19) ED	-		(6) CRD	(13) CLD	(6) CHD	-	(14) CLD	(17) CVD
**Others**	-	-	-	-	-	(10) Mal (6) CRD	-	-	-	(17) ND (17) BI (100) fracture patients
**≤5 %**	CHD, CRD, Mal, AD	Mal	CLD, CVD, CRD, CHD, Mal, IC	CRD, CHD, Mal	-	CHD, TB	-	-	-	-
**Radiological findings (%)**										
**Description ^°^**	-	-	(43) GGO	-	-	(24) PE	-	-	-	(100) GGO/ Cons
**Single (S)/Multiple (M) lobe lesions**	-	-	(42) M	-	-	(98) M	(89) M (11) S	-	-	(100) M
***Distribution***										
**Unilateral (U)/ Bilateral (BI)**	-	-	(42) Bi (11) U	-	-	(98) Bi	(11) U	-	-	-
**Central (C) / Peripheral (P)/ Both**	-	-	-	-	-	-	-	-	-	-
**Outcome No. (%)**										
**Healed/Discharged**	91 (27)	87 (60)	31 (23)	67 (100)	-	-	17 (95)	-	-	-
**Hospitalized**	183 (54)	-	13 (10) ^°°^	-	-	-	-	-	-	2 (33)
**Death**	65 (19)	27 (24)	-	-	51 (100)	19 (35)	1 (6)	-	-	4 (67)
**Days of Hospital Stay Duration - Median (IQR)**	28 (15-28)	-	-	-	-	12 (1-45)	-	-	-	-

	Niu S et al. ^a^ [19]	Kerpel A et al. [20]	Graselli G et al. [21]	Zhu T et al. [22]	Chen Z et al. [23]	Guo T et al. [24]	^**^Sun H et al. [25]	^**^Sun H et al. [25]	Bruno G et al. [26]	Gold J et al. [27]
**Publication Month - 2020**	Apr	Apr	Apr	Mar	Mar	May	May	May	May	May
**Country**	China	Israel	Italy	China	China	China	China	China	Italy	USA
**No. Patients ≥60 Years Old**	60	3	961	28	20	105	123	121	31	117
**Age**	≥65	60-62	≥61	>60	≥60	≥ 60	≥ 60	≥ 60	≥65	≥65
**Male No.**	34	3	783	16	-	48	51	82	19	-
**Symptoms^+^ (%)**										
	(79) Fev	(100) Fev	-	(86) Fev	-	(67) Fev	(85) Fev	(89) Fev	(81) Fev	-
	(57) Cgh	(67) Cgh	-	(47) Cgh	-	(65) Cgh	(74) Cgh	(73) Cgh	(81) Cgh	-
	(30) Dysp	(67) Ft/Mya	-	(33) Ft/Mya	-	(33) Ft	(39) GiS	(33) GiS	(48) Dysp	-
	(24) Ft/Mya	(33) NC	-	(29) Dysp	-	(30) Dysp	(29) Dh	(30) Dh	(19) Ft	-
	(7) Hdc	(33) Syn	-	(22) Dh	-	(10) Dh	-	-	-	-
		-	-	(22) AP	-	(8) Mya	-	-	-	-
**Others**	-	-	-	-	-	(6) N/V	-	-	-	-
**≤5 %**	-	-	-	-	-	Hdc, Anx	AP	AP	GiS, AP	-
**Comorbidities^++^ (%)**										
	(48) HTN	(67) HTN	(38) HTN	-	-	(44) HTN	(55) HTN	(63) HTN	(87) HTN	(86) HTN
	(29) CLD	(67) Smo	(18) CVD	-	-	(26) DM	(20) DM	(22) DM	(26) Smo	(47) CVD
	(16) CVD	(33) DM	(16) HC	-	-	(16) CVD	(12) CVD	(17) CLD	(16) CLD	(44) DM
	(10) DM	(33) CVD	(14) DM	-	-	(12) Smo	-	(17) CVD	(16) CVD	(21) CKD
	(6) CBD	(33) PS	(8) Mal	-	-	(9) CLD	-	-	(13) DM	(19) CLD
**Others**	-	(33) DL	(15) NS	-	-	-	-	-	(9) CKD	-
**≤5 %**	-	-	CLD, CRD, CHD	-	-	CLD, CKD	CLD, CBD	-	Mal	Smo, Mal
**Radiological findings (%)**										
**Description ^°^**	-	(67) Cons	-	(89) GGO/ Cons (54) GGO (22) Cons (7) PE	-	(40) GGO	-	-	(65) GGO/Cons (32) Cons	-
**Single (S)/Multiple (M) lobe lesions**	-	(100) M	-	(93)M (7) S	(65)M	(89)M	-	-	-	-
***Distribution***										
**Unilateral (U)/ Bilateral (BI)**	-	(100) Bi	-	-	(65) Bi (35) U	(89) Bi (1) U	-	-	(96) Bi (3) U	-
**Central (C) / Peripheral (P)/ Both**	-	(67) P	-	(60) P (36) Both	-	-	-	-	-	-
**Outcome No. (%)**										
**Healed/Discharged**	23 (38)	1 (33)	111 (12)	-	-	90 (86)	123 (100)	-	7 (23)	65 (56)
**Hospitalized**	32 (53)	2 (67)	525 (55) ^°°^	-	-	12 (11)	-	-	18 (58)	16
**Death**	5 (9)	-	322 (34)	-	-	3 (3)	-	121 (100)	6 (19)	36 (31)
**Days of Hospital Stay Duration - Median (IQR)**	-	-	-	-	-	17 (13-23)	-	-	23 (16-30)	10 (6-16)

* Cheng B et al. describes two separate populations (separated according to outcome) within *same* study.

** Sun H et al. describes two separate populations (separated according to outcome) within *same* study.

a Niu S et al. recorded the comorbidities of only 31/60 of their patients.

Abbreviations

+ Symptoms: Fev; Fever, Cgh; Cough, Ft; Fatigue, Mya; Myalgia, Ft/Mya; Both Fatigue and Myalgia, NC; Nasal Congestion/Runny nose, Hdc; Headache, SP; Sputum Production/Expectoration, ST; Sore Throat, Dysp; Dyspnea, CT; Chest Tightness, Dh;Diarrhea, N; Nausea, V; Vomit, N/V; Both Nausea and Vomit, Diz; Dizziness, Anx; Anorexia , Pha; Pharyngalgia, Hp; Hemoptysis, AG; Abnormal Gait, Con; Confusion, CP; Chest Pain, AP; Abdominal Pain, Del; Delirium, Chi; Chills, Syn; Syncope, GiS; GI Symptoms

++ Comorbidities: HTN; Hypertension, DM; Diabetes, CLD; Chronic Lung Disease, CVD; Cardiovascular Disease, CRD; Chronic Renal Disease, CHD; Chronic Hepatic Disease, Mal; Malignancy (active or in remission), Smo; Smoking history, AD; Autoimmune Disease, CBD; Cerebrovascular Disease, ED; Endocrine Disease, PS; Past Surgery, IC; Immunocompromised, TB; Tuberculosis, ND; Neurological Disorder, OP; Osteoporosis, BI; Brain Injury, CD; Collagen Disease, NS; Not Specified, DL; Dyslipidaemia, HC; Hypercholesterolemia *(Radiological Findings)*

° Description: GGO; Ground Glass Opacity, Cons; Consolidation, GGO/Cons; Both GGO and Cons, PE; Pleural Effusion *(Outcome)*

°° ICU (hospitalized)

### Study Characteristics

The majority of the studies were published during March and April 2020 and included the countries China (14/20), Italy (2/20), USA (2/25), France (1/20), and Israel (1/20). Seven articles revolved solely around COVID-19 patients older than 59 years old, and as such were included in total [10,11,13,16,24-26]. Chang B et al and Sun H et al described the discharged and deceased older COVID-19 patients separately within their respective studies (Cheng B: 67 discharged vs 51 deceased, Sun H: 123 discharged vs 121 deceased) [13,25]. In order to allow for better insight, both populations from both studies were included, yet their symptoms, comorbidities, outcome, and laboratory findings were kept separated. As for the rest of the studies (13/20) [12,14,15,17-23,27-29], careful assessment and data extraction of information pertaining to ≥60 years old COVID-19 patients were required. All of the studies made clear mention or distinction of the age of patients in regard to data. In total, 4965 older individuals with SARS-CoV-2 infection were identified and included.

**Table 2:** Laboratory Findings.

	Wang L et al. [10]	Zhu X et al. [11]	Lian J et al. [12]	^*^Cheng B et al. [13]	^*^Cheng B et al. [13]	Chen T et al. [14]	Liu K et al. [15]	Godaert L et al. [16]
**Publication Month - 2020**	Mar	Mar	Mar	Apr	Apr	Apr	Mar	Apr
**Country**	China	China	China	China	China	China	China	France
**No. Patients ≥60 Years Old**	339	114	136	67	51	55	18	17
**Age**	>60	>70	≥60	≥60	≥60	≥65	≥60	>67
**Male**	166	67	58	22	31	34	12	8
**Symptoms No.**	Table 1	Table 1	Table 1	Table 1	Table 1	Table 1	Table 1	Table 1
**Comorbidities No.**								
**Outcome No.**								
**Laboratory Findings - Median (IQR)**	Cheng B. et al presented results as Mean with SD							
**WBC Count (x10^9^/L)**	5.74 (4.37-8.29)	6.67 (4.56-9.05)	4.8 (3.9-6.4)	-	-	6.1 (2-25.7)	5.38 (4.33-6.13)	-
**Lymphocyte Proportion %**	-	-	-	-	-	-	19.15 (10.58-26.93)	-
**Neutrophil Proportion %**	-	-	-	-	-	-	68.3 (63.01-78.13)	-
**Neutrophil Count (x10^9^/L)**	4.43 (2.76-6.62)	5.19 (3.101-7.803)	3.2 (2.5-4.4)	3.9+/-2.1	6.5+/-4.8	4.7 (1-21)	-	4 x (<4.0)
**Lymphocytes Count (x10^9^/L)**	0.90 (0.59-1.29)	0.805 (0.55-1.17)	1.1 (0.7-1.4)	1.2+/-0.6	0.6+/-0.3	0.89 (0-6.0)	-	13x (<1.5)
**ESR (mm/h)**	-	-	-	39.7+/-25.7	56.6+/-33.9	45 (9-96)	-	-
**C-reactive Protein (CRP) (mg/L)**	49.6 (18.5-93.2)	59.1 (20.2-110.6)	19.0 (5.6-44.7)	30.8+/-37.1	123.5+/-109.0	85.3 (2-284)	22.74 (13.33-34.82)	16x(>10)
**Interleukin-6 (IL-6) (pg/mL)**	10.9 (5.2-25.4)	-	-	-	-	1.4 (0-200)	-	-
**Procalcitonin (ng/mL)**	0.08 (0.04-0.17)	0.104 (0.053-0.315)	-	-	-	0.48 (0-8)	0.04 (0.02-0.09)	-
**Albumin (g/L)**	-	33.85 (31.7-37)	39.2 (36.0-42.0)	38.6+/-4.9	33.1+/-5.0	33.3 (25-43)	33 (29-38)	-
**Hemoglobin Hb (g/L)**	121 (109-130)	-	129.0 (120.3-140.8)	-	-	-	107.35 (92.13-120.35)	-
**Platelet Count (x109/L)**	205 (151-259)	-	169.5 (132.0-207.5)	-	-	162 (22-414)	207 (159-287)	7x(<15)
**Lactate dehydrogenase (LDH) (U/L)**	301 (224-429)	328.5 (224-447)	244.0 (206.0-311.0)	262.0+/-249.8	453.4+/-266.8	395 (153-775)	-	-
**APTT (s)**	28.5 (26.2-31.3)	-	-	-	-	-	-	-
**PT (s)**	12.1 (11.6-12.7)	-	-	-	-	-	-	-
**D-Dimer (mg/L)**	1.20 (0.62-3.25)	1.5 (0.725-5.465)	-	0.95+/-1.35	4.59+/-7.13	1.35 (0.108-18.83)	-	-
**Cardiac Troponin (CtnI) (ng/mL)**	0.010 (0.006-0.030)	-	-	-	-	-	-	-
**Creatine Kinase (CK) (U/L)**	63 (40-104)	66.5 (42-130)	74.5 (52.3-123.0)	88.6+/-56.7	287.3+/-500.4	141.2 (13-972)	-	-
**BUN (Blood Urea Nitrogen) (mmol/L)**	-	7.2 (4.92-9.7)	4.4 (3.6-5.9)	6.4+/-6.1	9.1+/-5.2	-	-	-
**Creatinine (μmol/L)**	61 (50-76)	68 (57-96)	69.0 (58.6-79.6)	123.0+/-218.8	141.4+/-166.2	104.2 (37-787)	106.44 (79.83-150.79)	-
**Alanine aminotransferase (ALT) (U/L)**	27 (17-44)	32 (22-48)	21.0 (16.0-29.0)	25.1+/-19.0	32.4+/-32.7	41.3 (8-279)	-	-
**Aspartate aminotransferase (AST) (U/L)**	32 (23-46)	32 (22-48)	28.0 (22.0-36.0)	-	-	63.1 (18-209)	-	-

	Liu Y et al. [17]	Mi B et al. [18]	Guo T et al. [24]	^**^Sun H et al. [25]	^**^Sun H et al. [25]	Bruno G et al. [26]	Yuan Y et al. [28]
**Publication Month - 2020**	Mar	Apr	May	May	May	May	Mar
**Country**	China	China	China	China	China	Italy	China
**No. Patients ≥60 Years Old**	7	6	105	123	121	31	4
**Age**	>61	>75	≥ 60	≥ 60	≥ 60	≥65	64-71
**Male**	4	2	48	51	82	19	2
**Symptoms No.**	Table 1	Table 1	Table 1	Table 1	Table 1	Table 1	4 Cough 2 Fever 2 Sputum Production 1 Sore throat
**Comorbidities No.**							4 <82 GNRI^**^ 1 HTN
**Outcome No.**							4 Discharged
**Laboratory Findings - Median (IQR)**								
**WBC Count (x10^9^/L)**	5.24 (4.65-6.3)	10.43 (7.61-12.10)	4.9 (3.8-6.5)	5.37 (4.43-6.70)	10.15 (6.20-13.41)	6.43 (4.68-8.89)	5.37 (4.355-6.405)
**Lymphocyte Proportion %**	19.8 (9.3-21)	-	-	-	-	-	35.85 (25.43-44.75)
**Neutrophil Proportion %**	74.8 (70-85.9)	-	-	-	-	-	50.9 (46.175-58.25)
**Neutrophil Count (x10^9^/L)**	3.73 (3.33-5.39)	8.77 (5.8-11.10)	3.6 (2.6-4.5)	-	-	-	2.755 (2.02-3.76)
**Lymphocytes Count (x10^9^/L)**	0.8 (0.51-0.87)	0.70 (0.5-1.03)	1.1 (0.7-1.4)	0.95 (0.70-1.34)	0.51 (0.35-0.72)	0.83 (0.61-1.19)	1.76 (1.61-1.83)
**ESR (mm/h)**	-	-	-	36.0 (20.0-59.0)	37.0 (20.0-60.0	-	-
**C-reactive Protein (CRP) (mg/L)**	52.95 (45.6-69.3)	25.95 (17.8-48.75)	33.6 (9.4-56.9)	39.8 (8.3-79.6)	105.4 (65.0-165.2)	79.2 (34.7-133.5)	4.7 (4.31-4.91)
**Interleukin-6 (IL-6) (pg/mL)**	-	-	-	12.7 (3.3-41.5)	75.2 (35.2-162.9)	-	-
**Procalcitonin (ng/mL)**	0.077 (0.044-0.167)	0.49 (0.22-1.95)	-	0.05 (0.03-0.09)	0.36 (0.14-1.01)	0.12 (0.07-0.18)	3x(<0.10) / 0.13
**Albumin (g/L)**	38.3 (35.95-40.55)	33.9 (33.5-36.33)	36.2 (33.3-39.1)	-	-	-	-
**Hemoglobin Hb (g/L)**	-	-	125 (115-136)	-	-	12.5 (11.7-14.2)	-
**Platelet Count (x109/L)**	119 (118-141)	153 (144-178)	171.0 (135.8-228.8)	-	-	193 (143-293)	-
**Lactate dehydrogenase (LDH) (U/L)**	662 (551.5-708)	-	208.8 (173.1-239.4)	-	-	268 (213.5-335)	180 (166.75-183.25)
**APTT (s)**	-	31.98 (31.34-34.68)	33.9 (30.0-37.9)	-	-	-	-
**PT (s)**	-	13.18 (12.39-13.78)	12.5 (11.4-13.1)	-	-	-	-
**D-Dimer (mg/L)**	-	12.45 (7.04-15.88)	0.6 (0.3-1.9)	0.79 (0.47-1.63)	4.16 (1.49-21.00)	-	73.5 (54.5-99.5)
**Cardiac Troponin (CtnI) (ng/mL)**	0.012 (0.012-0.012)	-	-	-	-	-	2 x (<0.010)
**Creatine Kinase (CK) (U/L)**	107.5 (84.25-307.5)	-	84.5 (53.1-143.4)	-	-	-	62 (50-66.5)
**BUN (Blood Urea Nitrogen) (mmol/L)**	5.37 (4.64-6.2)	8.74 (6.2-16.01)	5.5 (4.2-7.3)	-	-	2.8 (2.1-4.1)	-
**Creatinine (μmol/L)**	81.9 (50.3-113)	96 (80.5-128)	66.9 (55.0-80.0)	66 (54-82)	90 (76-118)	89.3 (67.2-108.76)	-
**Alanine aminotransferase (ALT) (U/L)**	26.6 (26.25-37.15)	15 (15-17.25)	19.8 (15.0-28.3)	22 (14-36)	28 (19-42)	-	-
**Aspartate aminotransferase (AST) (U/L)**	33.6 (26.45-47.05)	32 (27.5-37.25)	26.0 (20.9-33.9)	28 (20-39)	45 (30-64)	-	18.6 (16.675-23)

* Cheng B et al. are two separate populations within the same study. Laboratory values presented as means with standard deviations

** GNRI; Geriatric Nutritional Risk Index

### Symptoms

Seventeen studies reported symptom presentation, pertaining to 1285 elderly patients [10-22,24-26,28]. Fever and cough were the first and second most common symptoms respectively, in which fever presented in 83.6% and cough in 62.7%. To a lesser extent, dyspnea (25.5%), fatigue (19.9%), sputum production (17.7%), chest tightness (15.3%), and diarrhea (13.0%) were present in the patients. Moreover, 6 studies reported separate symptoms fatigue (19.9%) or myalgia (4.6%), while 10 studies reported them as combined (8.0) [10-19,20,22,24,26]. Similarly, nausea (0.4%) and vomiting (0.3%) were separated by Zhu X et al, while combined (4.4%) by the rest [10,11,13-15,17,18,24-26]. Anorexia was observed in 8.4% of the elderly population with COVID-19. Other symptoms, including neurological symptoms, may be found in Table 1 and summarized in Table 4.

### Comorbidities

Comorbidities of 2288 elderly patients from 16 studies were found [10-15,17-21,24-28]. Niu S et al. recorded the comorbidities of only 31/60 of their elderly population, and as such only those patients with recorded medical history were included in the total estimate for comorbidities in Table 4 [19]. The main comorbidities observed were hypertension (42.8%), cardiovascular disease (22.6%), and diabetes (17.3%). Hyper-cholesterolemia (6.8%), chronic lung disease (8.2%), and malignancy (4.7%) were also reported. One article (n=6) described the results of older COVID-19 patients with fractures [18]. Few other comorbidities with less than 5% incidence were also reported (Table 1, Table 4).

### Radiological Findings

Out of the 20 articles, only 9 reported radiological findings for the older individuals [12,14,15,18,20,22-24,26]. The nine studies totalled 402 patients. The most common description reported was ground glass opacities (GGO) (28.6%), or GGO + consolidations (12.9%), affecting multiple lobes (62.2%) in a bilateral distribution (58.2 %). Despite the majority of studies not specifying peripheral or central distribution (93.0%), the recorded cases seem to point to a peripheral tendency for the disease (4.5%) with some cases being both peripheral and central (2.5%). No cases of purely central distribution were reported. Pleural effusion was found in 15 (3.7%) patients, and normal radiological findings in 11 (2.7%) (Table 1, Table 4).

### Outcome

Outcome of 4531 older patients in 14 studies was recorded [10-12,14,15,18-21,24,26-29]. As per date of publication of individual articles, about half the patients were hospitalized (50.5%), which includes 23.5% in the ICU. Only a portion (26.6%) of the patients had been discharged, while the mortality rate of patients ≥60 years old in this review showed to be one in five (20.0%). A combined 131 patients (2.9%) had unspecified outcomes (Table 1, Table 4). Studies comparing data from solely dead vs discharged patients, were excluded from review estimation of mortality rate due to unclear total hospitalized elderly COVID-19 population [13,25]. Limited studies reported hospital stay duration [10,14,24,26,27,29]. The shortest median hospital stay duration was 4.5 days with the longest being 28 days [10,29]. Despite difficulty in assessing coherence due to limited data, the majority of studies reported a median hospital stay duration between 10 to 23 days [14,24,26,27].

**Table 3:** Complications and Treatment.

	Wang L et al. [10]	Zhu X et al. [11]	Lian J et al. [12]	Chen T et al. [14]	Liu K et al. [15]	Godaert L et al. [16]	Liu Y et al. [17]	Mi B et al. [18]	Graselli G et al. [21]	Yuan Y et al. [28]	Guo T et al. [24]	Bruno G et al. [26]	Gold J et al. [27]	Richardson S et al. [29]
**Publication Month - 2020**	Mar	Mar	Mar	Apr	Mar	Apr	Mar	Apr	Apr	Mar	May	May	May	Apr
**Country**	China	China	China	China	China	France	China	China	Italy	China	China	Italy	USA	USA
**No. Patients ≥60 Years Old**	339	114	136	55	18	17	7	6	961	4	105	31	117	2582
**Age**	>60	>70	≥60	≥65	≥60	>67	>61	>75	≥61	64-71	≥ 60	≥65	≥65	>65
**Male**	166	67	58	34	12	8	4	2	783	2	48	19	-	-
**Symptoms No.**	Table 1	Table 1	Table 1	Table 1	Table 1	Table 1	Table 1	Table 1	Table 1	Table 2	Table 1	Table 1	Table 1	-
**Comorbidities No.**														-
**Outcome No.**														676 Discharged 1487 (613 ICU) Hospitalized 419 Death
**Hospital Stay duration (days) - Median (IQR)**														Discharged: 4.5 (2.7 - 7.2) Still hospitalized: 4.4 (2.3-8.0) Dead: 4.4 (2.1 - 7.1)
Complications^*^(%)	Most common complications of patients ≥60 years old														
	(42) SI	-	(17) ARDS	-	(39) HI	(59) RI	(100) Pn	(33) SI	-	-	(10) ARDS	(35) ARDS	-	(30) RI
	(29) HI	-	(10) RI	-	(39) RI	(47) HI	(86) ARDS	-	-	-	(5) AHI	(29) SI	-	-
	(21) ARDS	-	(10) HI	-	(22) SI	-	(43) RF	-	-	-	(5) RI	(10) Sh	-	-
	(21) AHI	-		-	(22) ARDS	-	(29) RI	-	-	-	(1) HI	(1) AHI	-	-
	(17) CI	-	-	-	(17) AHI	-	(29) HI	-	-	-	-	-	-	-
**Others**	(8) RI	-	-	-		-	(29) SI (14) CI (14) Sh	-	-	-	-	-	-	-
**≤5 %**	Sh		Sh		Sh									HI
Treatment^**^ (%)														
	-	(97) AvT	(86) AvT	(62) Ster	(89) LR	-	(100) Rv	(100) AvT	-	(75) LR	(93) AvT	(68) AbT	-	-
	-	(85) AbT	(50) IFN	(27) IFN	(67) AbT	-	(100) IFN	(100) AbT	-	(75) Ig	(60) AbT	(61) LR	-	-
	-	(45) Ig	(31) Um	(18) LR	(33) IFN	-	(57) Ig	(50) Ster	-	(50) Um	(49) Ster	(55) Ster	-	-
	-	-	(18) Ig	(13) Um	(22) Ig	-	(57) Os	(33) Ig	-	(50) Cef	(43) Ig	-	-	-
	-	-	(9) LR	(9) Ig		-	(43) Ster		-	(50) Moxi	-	-	-	-
	-	-	-	-	-	-	-	-	-	(25) Os	-	-	-	-
	-	-	-	-	-	-	-	-	-	(25) IFN	-	-	-	-
	-	-	-		-	-	-		-	(25) Lin	-	-	-	-
**Supplementary Oxygen No. (%)**	-	104 (91)	-	49 (89)	17 (95)	8 (47)	-	6 (100)	615 (64)	-	89 (85)	28 (90)	104 (89)	-
**Mechanical Ventilation (MV) ^°^ No. (%)**	-	-	-	24 (44)	-	-	6 (86)	-	-	-	-	-	-	-
***Invasive MV***	-	-	6 (5)	-	4 (22)	-	3 (43)	0	709 (74)	-	8 (8)	8 (26)	48 (41)	558 (22)
***Non-Invasive MV***	-	-	3 (2)	-	1 (6)	-	-	3 (50)	80 (8)	-	7 (7)	-	5 (4)	-
**Other Treatments^°°^ (%)**	-	(70) TCM (50) HT (28) VD (3) Hem	-	(55) MA (9) ThyF	(78) ThyP (78) TCM (11) RRT (6) ECMO	-	-	-	-	(75) ThyF (75) Chlor (75) S	(64) NCa (3) ECMO	(77) HyC (74) Hep (13) Toc	(82) NCa (11) RRT (43) VS (7) CPR	(4) RRT

Abbreviations

* Complications: Pn; Pneumonia, CI; Cardiac Insufficiency, ARDS; Acute Respiratory Distress Syndrome, AHI; Acute Heart Injury, RI; Renal Impairment/Injury, HI; Hepatic Impairment/Injury, SI; Secondary Infection, Sh; Shock, RF; Respiratory Failure, MDS; Multiorgan Dysfunction Syndrome

** Treatment: AvT; Antiviral Therapy (not specified), LR; Lopinavir & Ritonavir, Um; Umifenovir, Fav; Favipiravir, IFN; Interferon, Ig; Immunoglobulin, Os; Oseltamivir, Rv; Ribavirin, Ster; Glucocorticoids/Corticosteroids, AbT; Antibiotic Therapy (not specified), Azi; Azithromycin, Cef; Ceftriaxone, Moxi; Moxifloxacin, Lin; Linezolid, HyC; Hydroxychloroquine, Hep; Heparin, Toc; Tocilizumab, NCa; Nasal Cannula, RRT; Renal Replacement Therapy, VS; Vasopressor Support, CPR; CPR

° MV that was not specified; invasive or non-invasive

°° Other Treatments: HT; Hormone Therapy, VD; Vasoactive Drugs, TCM; Traditional Chinese Medicine, Hem; Hemodialysis, ThyF; Thymalfasin, MA; Mucoactive agent/Expectorant, ThyP; Thymopentin, ECMO; Extracorporeal Membrane Oxygenation, RRT; Renal Replacement Therapy, Chlor; Chloroquine, SA; Serum Albumin, PPI; Proton Pump Inhibitor

### Laboratory Findings

The laboratory findings of 1194 patients were reported [10-18,24,26-28]. The majority of the results were presented as median (IQR), except for Cheng B et al. as mean and SD [13]. Lymphopenia (<1.1 x 10^9^/L) was observed in a considerable amount of the studies, as was occasional thrombocytopenia (<150 x 10^9^/L). Moreover, the median/mean C-reactive protein and ESR were evidently elevated in the patients, with majority reporting levels >10 mg/L and >35 mm/h respectively. Only 3 studies reported IL-6 levels [10,14,25]. Sun H and colleagues found elevated IL-6 levels (median of 75.5 with an IQR of 35.2-162.2) in the deceased elderly patients [25]. Few studies also reported higher levels of LDH (>300 U/L) and D-dimers (>1.0 mg/L). Hepatic and cardiac markers (ALT/AST and Cardiac Troponin) were mainly within normal range, while some studies showed slightly abnormal renal markers (Creatinine and BUN). Additional laboratory findings may be found in Table 2.

### Complications

Despite only 9 studies reporting complications, the results pertained to 3241 patients [10,12,15-18,24,26,29]. Renal impairment/injury was the most prominent complication with an incidence rate of 25.7%. The second and third most commonly reported complications were co-infection (4.9%) and hepatic impairment/injury (4.5%). Moreover, roughly 126 patients (3.9%) developed ARDS according to the data. Cardiovascular related complications, such as acute heart injury (2.4%), cardiac insufficiency (1.8%), and arrhythmia (1.1%) were also reported (Table 3, Table 4).

**Table 4:** Summary Table.

	No. Studies with reported results	Total patients			
**Symptoms**	16	1285		No. Patients	%
			*Fever*	1074/1285	83.6
			*Cough*	806/1285	62.7
			*Dyspnea*	328/1285	25.5
			*Fatigue*	256/1285	19.9
			*Sputum Production*	227/1285	17.7
			*Chest Tightness*	196/1285	15.3
			*Diarrhea*	167/1285	13.0
			*Anorexia*	108/1285	8.4
			*Fatigue & Myalgia*	103/1285	8.0
			*Myalgia*	59/1285	4.6
			*Nausea & Vomiting*	57/1285	4.4
			Others^*^	265/1285	20.6
**Comorbidities**	16	2288		No. Patients	%
			*Hypertension*	979/2288	42.8
			*Cardiovascular Disease*	517/2288	22.6
			*Diabetes*	395/2288	17.3
			*Chronic Lung Disease*	188/2288	8.2
			*Hypercholesterolemia*	156/2288	6.8
			*Malignancy (Active or in Remission)*	108/2288	4.7
			*Chronic Renal Disease*	83/2288	3.6
			*Cerebrovascular Disease*	54/2288	2.4
			*Chronic Hepatic Disease*	38/2288	1.7
			*Not Specified*	145/2288	6.3
			Others^**^	156/2288	6.8
**Radiological Findings**	9	402	Description	No. Patients	%
			*Ground Glass Opacity (GGO)*	115/402	28.6
			*GGO + Consolidations*	52/402	12.9
			*Pleural effusion*	15/402	3.7
			*Consolidations*	18/402	4.5
			*Normal*	11/402	2.7
			**Distribution**		
			*Multiple Lobes*	250/402	62.2
			*Single Lobe*	4/402	1.0
			*Not Specified*	148/402	36.8
			*Bilateral*	234/402	58.2
			*Unilateral*	32/402	8.0
			*Not Specified*	136/402	33.8
			*Peripheral*	18/402	4.5
			*Peripheral + Central*	10/402	2.5
			*Central*	0/402	0.0
			*Not Specified*	374/402	93.0
**Outcome**	14	4531		No. Patients	%
			*Hospitalized (*As of date of publication)	2290/4531	50.5
			*Discharged*	1203/4531	26.6
			*Death*	907/4531	20.0
			*Not Specified*	131/4531	2.9
**Complications**	9	3241		No. Patients	%
			*Renal Impairment/ Injury*	832/3241	25.7
			*Secondary Infection*	160/3241	4.9
			*Hepatic Impairment/ Injury*	147/3241	4.5
			*ARDS*	126/3241	3.9
			*Acute Heart Injury*	79/3241	2.4
			*Cardiac insufficiency*	59/3241	1.8
			*Arrhythmia*	35/3241	1.1
			*Others ^+^*	57/3241	1.8
**Treatment (drugs)**	9	476		No. Patients	%
			*Antiviral Therapy^++^*	330/476	69.3
			*Antibiotic Therapy-*	199/476	41.8
			*Immunoglobulin*	134/476	28.2
			*Glucocorticoids*	107/476	22.5
			*Interferon*	98/476	20.6
			*Lopinavir & Ritonavir*	61/476	12.8
			*Umifenovir*	49/476	10.3
			*Oseltamivir*	5/476	1.1
			*Ribavirin*	4/476	0.8
**Supplementary Oxygen**	9	1424		1020/1424	71.4
**Treatment (Mechanical Ventilation)**	10	4018		No. Patients	%
			*Invasive MV*	1344/4018	33.4
			*Non-Invasive MV*	96/4018	2.4
			*MV - Not specified*	30/4018	0.7

* (Symptoms) Others: include Unspecified GI symptoms, Headache, Dizziness, Sore Throat, Pharyngalgia, Abdominal Pain, Delirium, Nasal Congestion, Abnormal Gait, Chills, Confusion, Nausea, Vomiting, Hemoptysis, Chest Pain, Syncope

** (Comorbidities) Others: include Smoking history, Autoimmune disease, Endocrine Disorders, Past Surgery, Tuberculosis, Immunocompromised, Neurological Disorder, Osteoporosis, Brain Injury, Fractures, Geriatric Nutritional Risk Index (GNRI) <82, Dyslipidemia, Alzheimer, BMI >29.

+ (Complications) Others (%): include Pneumonia, Acute Liver & Kidney injury, Shock, Respiratory Failure, Multiorgan Dysfunction Syndrome (MODS), Sepsis, Allergic eruption, Pneumothorax, Pancreatitis

++ (Treatment - Drugs) Antiviral Therapy: Not specified, - Antibiotic Therapy: Not specified

### Treatment

In order to better reflect the current use of therapeutic approaches, in the summary table (Table 4) treatment was separated into studies which documented relevant drugs used (9 studies - 476 patients) [11,12,14,15,17, 18,24,26,28], studies with mention to supplementary oxygen use (9 studies - 1424 patients) [11,14-16, 18,21,24,26,27], and studies with announced information concerning mechanical ventilation (10 studies - 4018 patients) [12,14,15,17,18,21,24,26,27,29]. Treatment approaches implemented in less than 5% of patients can be observed in Table 3.

Unspecified antiviral use was the most commonly reported therapeutic approach (330/476-69.3%). Lopinavir/Ritonavir was the most common type of antivirals used (12.8%), followed by Umifenovir (10.3%), and some cases documented the use of Oseltamivir (1.1%) and Ribavirin (0.8%). Unspecified antibiotics were also amongst the most common treatments administered (41.8%). Almost twenty to thirty percent of patients additionally received interferon therapy (20.6%), glucocorticoids (22.5%), and supplementary immune-globulins (28.2%). All, except 1 study, reporting drug treatment originated in China.

From 9 studies that recorded the use of supplementary oxygen, 1020 older patients out of 1424 (71.6%) required the administration of inhalant oxygen [11,14-16,18,21,24,26,27]. 7 out of the 9 studies recorded the need for supplementary oxygen in >80% of their elderly COVID-19 population [11,14,15,18,24,26,27]. A concise breakdown per study can be observed in Table 3.

Mechanical ventilation data was additionally extracted into Table 3 and summarized in Table 4. Roughly one in three (33.5%) older patients with reported respiratory status (10 studies - 4018 patients) required invasive mechanical ventilation (MV) [12,14,15,17, 18,21,24,26,27,29]. Non-invasive MV was recorded to a lesser extent in 2.4% of the patients. Other notable treatments, including Renal Replacement Therapy (RRT) and Traditional Chinese Medicine, was implemented by <5% of patients and can be referenced in Table 3.

### Qualitative synthesis

The summarized review findings and their qualitative confidence levels, presented as per CERQual approach, can be found in Table 5. The studies were mainly observational and descriptive in nature. Methodological limitations were noted to varying extents, amongst which selection bias was the most prominent due to 8 studies admitting mainly serious cases, including 1 study that solely revolved around ICU patients [10,11,14,15,21,25-27]. The data was the thinnest in relation to radiological findings and current use of treatment. Due to urgency of the current situation, the majority of studies reported a short follow up duration of <1 month. As such, an estimated 50.5% of the patients were still hospitalized as of date of publication. Such limitations may hinder the accuracy of outcome data (mortality rate) and complications. The highest coherence in results was found in symptoms and comorbidities, followed by laboratory findings. With 14/20 studies originating from 1 geographic region (China), there are concerns for lack of international representation of data, especially pertaining to current use of treatments. The combination of short duration of studies, novelty of subject, large hospitalized population, and studies being largely from homogenic regions, no high confidence was allocated.

**Table 5:** CERQual assessment of Review Findings.

Review Finding	CERQual Assessment of Confidence in the Evidence	Explanation of CERQual Assessment	Studies Contributing to the Review Finding
***Symptoms***			
**Most Common:** Hospitalized older COVID-19 patients were commonly reported to experience fever or cough, and to a lesser extent dyspnea, fatigue (with or without myalgia), sputum production, chest tightness, or diarrhea (in order from most to least commonly reported).	Moderate	6 studies with minor to moderate methodological limitations, of which selection bias due to admission of severe patients was noted. Relatively adequate data pertaining to 1285 older COVID-19 patients. 13 studies originating in China, which leads to moderate concerns of lack of geographic diversity in reported data. High coherence.	10-18, 19, 20, 22, 24-26
**Less Common:** Less commonly observed symptoms in the elderly COVID-19 population included: headache, sore throat, GI symptoms, dizziness, delirium, nasal congestion, chills, chest pain, pharyngalgia, abnormal gait, syncope, nausea and vomiting (in no particular order).			
***Comorbidities***			
**Most Common:** Hypertension is the most commonly reported comorbidity in elderly COVID-19 patients, followed by cardiovascular disease, diabetes, chronic lung disease, and hypercholesterolemia.	Moderate	9 studies with minor to moderate methodological limitations. 2 studies had incomplete medical history of relevant patients, and 1 study revolved around COVID-19 presentation specifically in fracture patients. In total, adequate data, pertaining to 2288 patients, with reported comorbidities was present. Notably, smoking history was under-reported. Despite a predominance of studies from China, almost half of patients contributing to comorbidity conclusion stemmed from Italy, USA, and Israel. High coherence.	10-15, 17- 21, 24-28
**Less common:** Minority of studies reported patients with chronic renal, hepatic, cerebrovascular disease or malignancy. Elderly COVID-19 patients with a variety of other immune, endocrine, nutritional, and neurological comorbidities were also occasionally reported.			
***Radiological Findings***			
**Description:** Ground Glass Opacities (GGO) was the most commonly reported observation in radiological imaging of older COVID-19 patients. Also, isolated consolidations or in combination with GGO were, to a lesser extent, documented with occasional cases of pleural effusion. Few patients demonstrate normal imaging findings.	Low	4 studies with minor to moderate methodological limitations. Gaps and inconsistency in reporting of radiological findings was observed. Inadequate data, pertaining to only 402 elderly COVID-19 patients. Moreover, only 2 studies originating from regions other than China (Israel and Italy), which raises concern for lack of geographic diversity. Insufficient reported radiological findings clouds coherency judgement, however reasonable coherence can be seen from preliminary data.	12, 14, 15, 18, 20, 22-24, 26
**Distribution:** Adults ≥60 years old experience multiple lobe involvement in a bilateral distribution mainly. With primary data showing peripheral tendency of COVID-19.			
***Outcome***			
Almost half of total patients were still hospitalized as of date of individual study publication. However, hospitalized elderly COVID-19 patients with clear outcome show an evident risk for mortality. Majority of studies show an estimated mortality rate of >15%, with the total combined mortality rate being close to 20%. Hospital stay duration ranges from a few days to few weeks, with a common median of hospitalization being >10 days.	Low	14 studies with minor to significant methodological limitations. Possible selection bias due to hospital admission of mainly serious cases was noted in 7 studies, and in 1 study involvement of strictly ICU patients was observed. The dynamic nature of the situation led to short follow up time by majority of studies, which raises concerns for inadequacy of data. Reasonable coherence.	10-12,14,15, 18 - 21, 24, 26-29
***Laboratory findings***			
Lymphopenia (<1.1 x 10^9^/L) and elevated inflammatory markers, CRP (>10 mg/L) and ESR (>35 mm/h), are commonly observed in the elderly COVID-19 patients. Occasionally, thrombocytopenia (<150x10^9^/L), higher levels of LDH (>300 U/L), D-dimers (>1.0 mg/L), and renal markers (creatinine & BUN) can be seen. Other markers indicating organ damage, such as hepatic or cardiac, are mainly within normal range. IL-6 was an underreported biochemical variable.	Moderate	6 studies with minor to moderate methodological limitations. Minor concern for underlying comorbidities, baseline health, and associated medication use influence on results in 3 studies. Relatively adequate data pertaining to 1194 elderly COVID-19 patients. Overwhelming majority of data stemming from one geographic region (China), raises concern for lack of diversity. Moderate to high coherence.	10-18, 24-26, 28
***Complications***			
Besides a risk for secondary infection or ARDS, older COVID-19 patients are prone to renal injury over the course of the disease. Hepatic injury and cardiovascular related complications (including cardiac insufficiency or arrhythmia) can be observed to a lesser extent.	Low	7 studies with minor to significant methodological limitations. 3 studies admitted mainly severe cases of COVID-19, raising concern for potential selection bias. 6 studies had short observation time (<1 month), and large proportion of patients still hospitalized. Despite relatively adequate patient sample with reported complications (3241 patients), inconsistency in the documentation detail of complication is observed. Reasonable coherence.	10, 12, 15-18, 24, 26, 29
***Treatments***			
**Drugs:** Antiviral therapy is the main treatment approach for elderly COVID-19 patients, of which Lopinavir, Ritonavir, and Umifenovir are most commonly used. This is usually in combination with antibiotics, immunoglobulins, glucocorticoids, or interferon therapy.	Very Low	5 studies with minor to significant methodological limitations. Inadequate data, with detailed treatment plans and use of medication only pertaining to 476 patients. Moreover, all studies, except 1, originated from China raising substantial concerns regarding data reflectiveness of different international treatment approaches. Reasonable coherence	11, 12, 14, 15, 17, 18, 24,26, 28
**Supplementary Oxygen:** Almost 4 in 5 hospitalized older COVID-19 patients require supplementary oxygen inhalation.	Moderate	6 studies with minor to moderate methodological limitations. 1 study revolved around ICU patients. Relatively adequate data pertaining to 1424 hospitalized elderly COVID-19 patients. Studies were predominantly from China, however considerable amount of total reported patients were from other regions such as Italy, USA, and France. Moderate to high coherence.	11, 14, 15, 16, 18, 21, 24,26,27
**Mechanical Ventilation:** 20% to 50% of severely affected elderly patients may require invasive mechanical ventilation.	Low	4 studies with moderate methodological limitations. 4 studies admitting mainly severe cases. Concerns for restricted mechanical ventilation use due to limitations of available resources or overflow of patients. Relatively adequate data (4018 patients) from 3 geographic regions (USA, Italy, China). Generally low coherence, however reasonable coherence is observed amongst studies with mainly serious cases.	12, 14, 15, 17, 18, 21, 29, 24,26,27

## DISCUSSION

As of date of publication, more than 7 million people have been infected by SARS-CoV-2 worldwide. The virus continues to spread, and it has become crucial for health professionals to familiarize themselves with disease presentation in different age populations, amongst which the older individuals are at high risk. To our knowledge, this is the first systematic review to focus strictly on COVID-19 patients ≥60 years old, in an attempt to comprehensively and concisely describe the clinical picture in this age group.

Due to the novelty of SARS-CoV-2 pandemic, the majority of studies, post selection criteria, mainly predominated patients from China. Studies from the USA, Israel, Italy, and France were also selected. 2 studies provided information on a more age homogenous group of patients (70 years in age and greater) [11,18] with the remaining 18 studies having a greater age distribution (60 years and greater) of elderly patients. Out of 20 studies, 18 specified the gender of the patients of which a clear majority was male (1442 male vs 804 female). This coincides with other reported studies, where a pattern of males being more prone to COVID-19 infections is observed [30].

According to our systematic review of literature, the older adult population experience a spectrum of disease presentation. A high coherence was observed between studies in terms of reported symptoms and their incidence. While a majority of older individuals presented with common symptoms such as fever and cough, approximately 15% to 25% of older individuals also present with concurrent respiratory related symptoms such as dyspnea, sputum production, or chest tightness. Various reports have compared these concurrent symptoms between older and younger populations, and some significant differences have been observed [12,14,15,19,22]. Older adults also presented a large variety of symptoms including fatigue, with or without myalgia, gastrointestinal symptoms (diarrhea, nausea/vomiting, abdominal pain), anorexia, headache, dizziness, and others. Wang L et al. found that presence of dyspnea, low lymphocyte count, or cardiovascular and lung comorbidities in the elderly population were all factors predictive of worse disease progression [10]. However, further research is required to analyze the association of such symptoms to outcome.

In general, the laboratory findings in the COVID-19 elderly population revealed lymphopenia, elevated inflammatory markers (CRP and ESR), as well as elevated LDH and D-dimers. Few studies also showed thrombocytopenia in the patients [14,16,17]. When compared to younger populations, Liu K and colleagues demonstrated that older COVID-19 patients (≥60 years old) had significantly lower lymphocyte proportion as well as significantly higher CRP levels [15]. Chen T et al. further demonstrated this difference by comparing 148 younger COVID-19 patients (<65 years old) to 55 elderly COVID-19 patients (≥65 years old) [14]. In their study, findings of lymphopenia and higher levels of CRP were present in older individuals, as well as significantly larger proportion of the elderly population experiencing elevated hepatic injury markers (ALT/AST), renal injury marker (creatinine), inflammatory markers (IL-6, procalcitonin, and ESR), LDH, and D-dimers. Such differences in biochemical markers in older adults, as compared to the young populations, illustrate the potential for more grievous organ damage caused by the SARS-CoV-2 infection. In a retrospective analysis of almost 120 older patients (≥60 years old), Cheng B et al. showed that monitoring levels of D-dimers, LDH, albumin, urea nitrogen, and NLR can be used to early recognize severe cases of older COVID-19 patients [13].

Hospital stay duration, explicitly for the older COVID-19 population, was reported by 5 out of 20 studies reviewed [10,14,24,26,27]. The shortest time for hospital stay was recorded at 4.5 days (2.7-7.2) with the longest stay duration of 28 days (15-28) before discharge. An important notion to consider is the origin of patients and their health status when admitted into the hospital. Patient symptoms ranged from mild to severe with certain studies focusing mainly on severe patients. As a result, collected hospital stay duration data may not reflect the true nature of COVID-19 infection and patient treatment response. Additionally, only 1 study provided information with respect to the duration from onset of symptoms to the negative test confirmation of RT-PCR, for confirmatory clearance of the COVID-19 infection [14]. Guo T and colleagues revealed a median of 18 days (14-25) from symptom presentation to a measure of negative presence of the viral infection, providing evidence for ethical discharge [24]. Even due to the low volume of studies providing RT-PCR COVID-19 detection and hospital stay duration information, preliminary case study reports do reflect a similar time period of disease onset and progression [31]. Furthermore, Yuan Y et al. described 4 dynamic cases of recovered elderly COVID-19 patients (tested negative on RT-PCR), who eventually shifted and tested positive for virus RNA again [28]. Such phenomena indicate the need for further research analyzing the possible propensity of certain older adults to experience remission, and its significance on prognosis. Studies isolating the older populations can help illuminate the progression of COVID-19 and its estimated timeline in the elderly population.

Radiological findings were under-reported in our included studies, raising concerns for inadequacy of data for conclusive findings. However, the primary data showed reasonable coherence, documenting mainly ground glass opacities (GGO), in some cases with consolidations, affecting multiple lobes in a bilateral distribution. Tendency for peripheral distribution and involvement of middle and lower lung was also described [20,22,23]. Studies by Liu K et al. and Chen Z et al. revealed that the older population, when compared to younger populations, demonstrate more prominent radiological changes [15,23]. It is unclear whether such differences can be attributed to the overall immunological fragility of the aged adults or perhaps be linked to use of certain concurrent medications.

The most common comorbidities amongst the elderly COVID-19 population were hypertension, cardiovascular disease, diabetes, chronic lung disease (such as COPD) and hypercholesterolemia. A high coherence was observed between studies, with data pertaining to over 2000 patients in total. Despite being the most commonly observed comorbidity, Schiffrin EL et al. explained that hypertension, and its associated therapeutic drugs (ACE inhibitors/ARBS), are yet to show an association with the SARS-CoV-2 infection [32]. Moreover, in a recent study by Mehra MR et al., it was shown that underlying cardiovascular disease, COPD, and current smoking were all associated with a higher mortality rate amongst hospitalized COVID-19 patients [33]. Their findings also concluded that hypertension, hyperlipidemia, and diabetes were not factors independently predictive of death in this disease. Additionally, Lippi G. and Henry BM estimated that patients with COPD were about five and a half times more likely to develop severe infection due to SARS-CoV-2 [34]. Given that comorbidities in older individuals act as detrimental prognostic factors, careful attention to underlying disorders and their association with SARS-CoV-2 infection, through up-to-date scientific literature, is necessary to avoid worse prognosis [35].

Due to novelty of the subject, combined with short follow up time, a significant proportion of the older COVID-19 patients were still hospitalized as of day of publication of studies. Such limitations lead to a restricted assessment of the full extent of complication development in the elderly population. From 9 included studies, [10,12,15-18,24,26,29] the distribution of complications revealed renal impairment/injury to be of highest prevalence, developing in almost 25% of older individuals with the SARS-CoV-2 infection. Subsequent complications included secondary infections, hepatic impairment/injury, ARDS, and cardiovascular related complications (acute heart injury, cardiac insufficiency, arrhythmia). A considerable variability amongst studies was noted, raising concerns for coherence and validity of findings. Currently, to the authors’ knowledge there is no supporting literature, with respect to COVID-19 mechanisms of injury on kidney or hepatic systems. However, some suggested mechanisms include the virus causing direct cellular damage, or perhaps injury due to a triggered cytokine storm [36]. Renal and hepatic complications can further exacerbate the clinical prognosis, leading to more complicated treatment plans, longer hospital stays, and higher chances for mortality [37-39]. Such detrimental consequences raise the need for further research on the association between the renal and hepatic systems with the SARS-CoV-2. Further understanding can allow for more effective preventative measures, and also provide a different perspective on COVID-19 multi-organ effect and its process in this age group.

Besides the inadequacy of data, eight out of the nine studies with reported treatment originated from 1 geographic region (China), resulting in extremely limited international representation data. Nonetheless, a large proportion of observed patients received antiviral therapy, of which Umifenovir and Lopinavir/Ritonavir were most commonly specified. Interferon, immunoglobulins, antibiotics, and glucocorticoids were also therapies being applied, usually in different combinations. Only one study reported the use of Chloroquine [28]. Keeping in mind the incidence of renal complications, a proportion of the elderly COVID-19 patients also received Renal Replacement Therapy (RRT). It was reported by Graselli et al. that 73.7% (709/961 patients) of older COVID-19 patients (≥61 years old) admitted to the ICU required mechanical ventilation [21]. On the other hand, when describing 5700 patients in New York city (of which 2582 were ≥65 years old), Richardson S. and colleagues had an estimated 21.6% (558/2582) of older individuals requiring invasive MV. The total proportion of the elderly population in our review that required invasive MV was 33.4% (1344/4018), however this includes ICU patients, and as such the numbers can be partially overestimating the general incidence [21].

The epidemiological history and mode of infection of the elderly patients may carry important clues to patterns of disease presentation and spread. Seven studies within this review included patients from the Wuhan province, China, however only 1 described the mode of virus contraction [18]. Additionally, six other studies characterized the epidemiological history of the older COVID-19 population, all within the region of China [12,17,19,23,28,24]. Direct or indirect contact with infected individuals from Wuhan, usually via family clusters, was the main mode of transfer in included patients. Mi B et al. described COVID-19 nosocomial infection of some older patients hospitalized for fracture [18]. Preliminary data from the United States has shown the vulnerability of old age/nursing homes as potential vectors for rapid COVID-19 infection and spread [40]. The person-to-person contact within the nursing home environment seems to provide ideal conditions for older adult’s infection. Implementation of protective measures, especially within the elderly population, may prove extremely beneficial in mitigating the severe progression of COVID-19 in the most vulnerable. More data is required from various geographic regions to further assess any association between mode of infection and COVID-19 development in the older population.

With the uninterrupted spread of the coronavirus, and many patients continuing to be hospitalized and battling the disease, collecting and accurately summarizing the outcomes in the elderly population becomes challenging. However, the preliminary numbers collected in this review, pertaining to 4531 patients, suggest that approximately one-in-five cases of COVID-19 in older individuals will lead to death. Similarly, few studies with considerable population sizes have also reported the range of mortality rate in the elderly population to be between 16% to 24% [10, 11, 29]. This is the most severe in any age group [14,15,21,29].

### Limitations

Several limitations were experienced throughout the construction of this systematic review. Due to the novelty of the subject matter, sampling of data was limited to a relatively short time frame (1^st^ January to 1^st^ June 2020) and mostly included data originating from homogenous populations in articles stemming from regions of China. Concerns of a possible bias may exist within data, reflecting certain demographic populations more than others. Language barrier and limited search tools can lead to the possibility of undetected studies and missing reports within our search criteria. Limited samples from other populations/country regions were found as of the date of conducting this review. Furthermore, the selected studies were observational (retrospective) in nature. Only hospitalized or older individuals with a definite outcome were analyzed in this review, this may lead to full clinical spectrum not being adequately represented. Moreover, reviewed studies may have given priority to reporting of more severe cases in the attempt to provide a clearer picture of the infection. Medical standards and economic conditions vary from region to region, this may further influence patient care and community outcomes. Finally, the vetting process required the removal of samples that had results merged with other age populations (individuals <60 years of age), resulting in the exclusion of a significant proportion of available data.

### Conclusion

The purpose of this systematic review was to summarize the general clinical picture of SARS-CoV-2 infection in the elderly population. A large variety of symptom presentations can be observed, including respiratory, gastrointestinal, cardiovascular and neurological manifestations. Abnormalities in inflammation related laboratory measures are also evident, and in some cases indicative of multi-organ involvement. Development of renal complications and to a lesser extent hepatic and cardiac complications should also be monitored. Further research is required to analyze possible patterns of disease presentation and effective treatment plans in older populations. Presented literature can assist in the construction of better predictive models of COVID-19 in older adults.
